# Supplementary figures and images for: Corollary discharge and efference copy mechanisms in schizophrenia and controls: The N1 and P2 evoked potential components differentially react to self-initiated tones in schizophrenia
Source: PLoS One. 2025 Dec 11;20(12):e0336046. doi: 10.1371/journal.pone.0336046 (PMC12698011; doi:10.1371/journal.pone.0336046)

**Statistical analysis of LRP:**
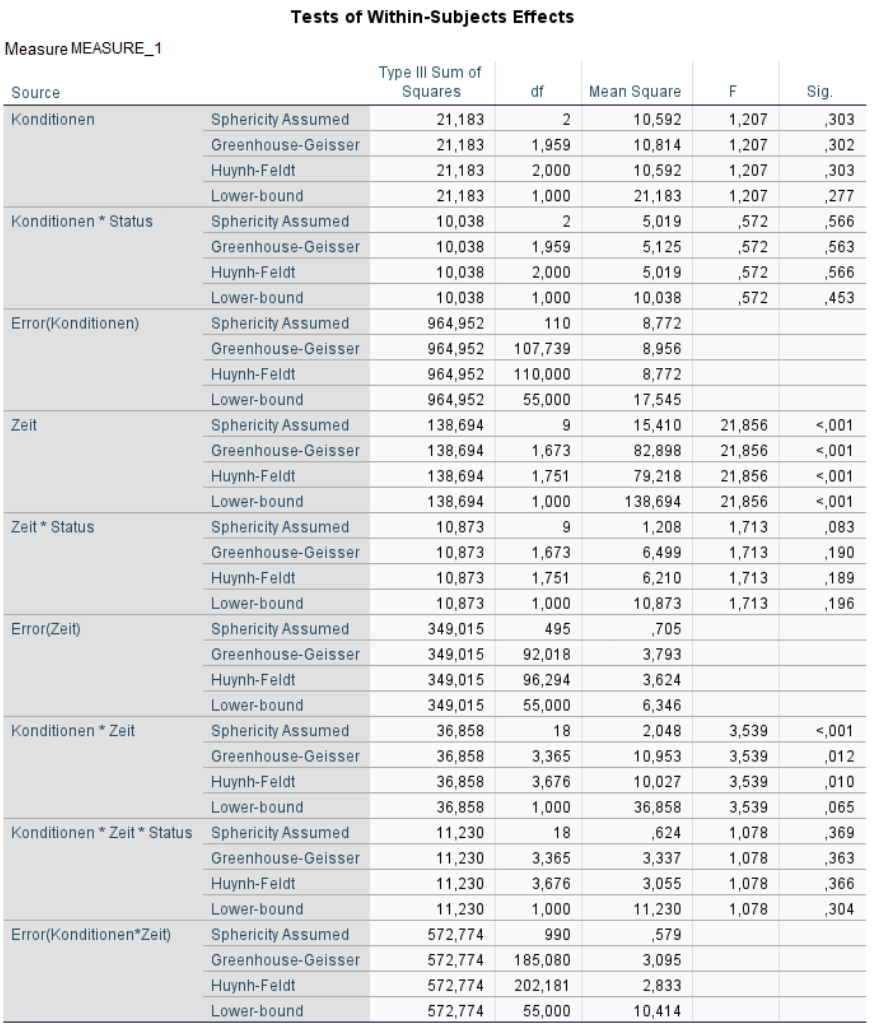


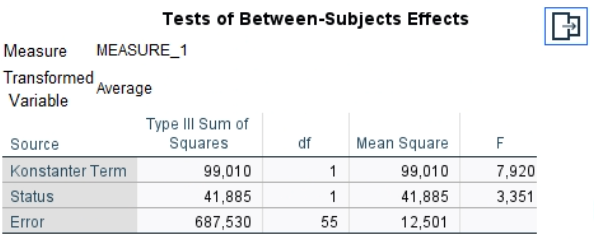


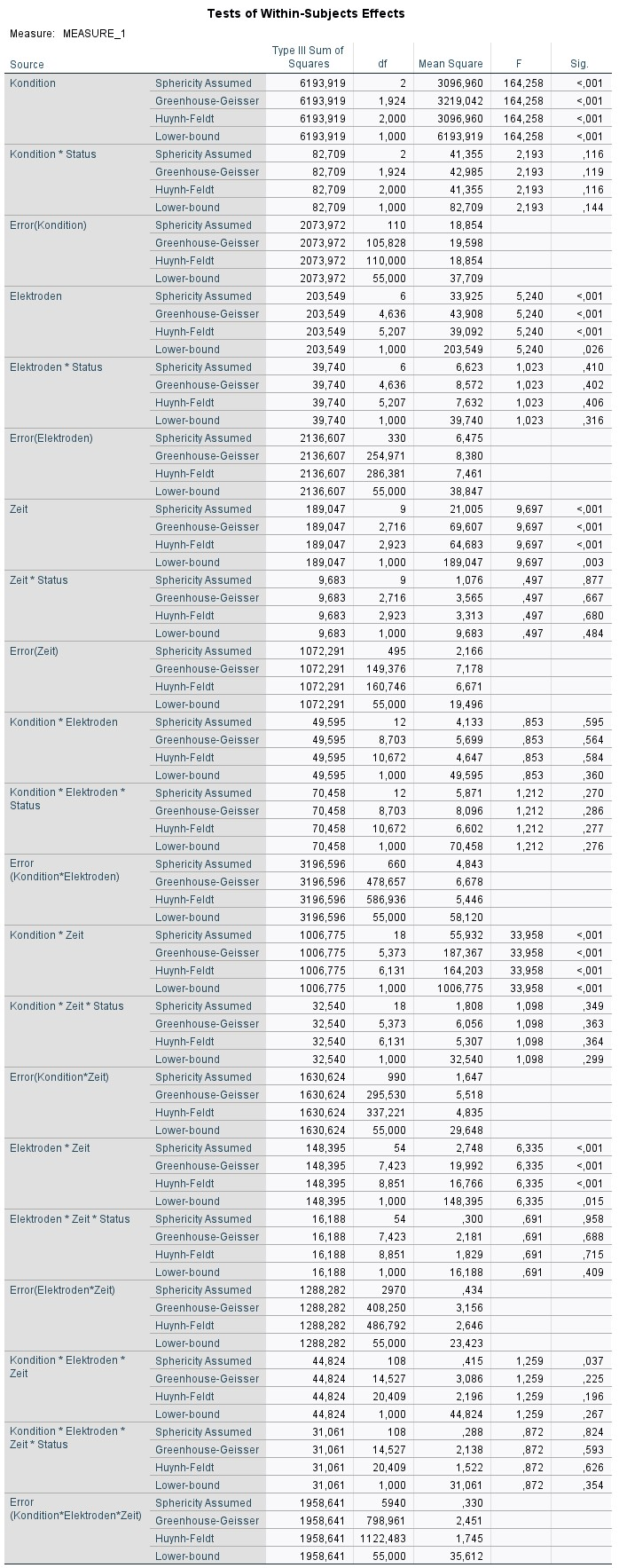


**Statistical analysis of RP:**


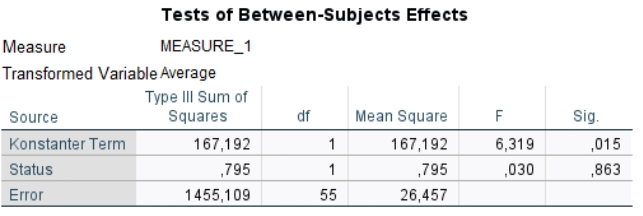

Supplement: S1 File — (DOCX) [file pone.0336046.s001.docx]
