# Supplementary material for: Corollary discharge and efference copy mechanisms in schizophrenia and controls: The N1 and P2 evoked potential components differentially react to self-initiated tones in schizophrenia
Source: PLoS One. 2025 Dec 11;20(12):e0336046. doi: 10.1371/journal.pone.0336046 (PMC12698011; doi:10.1371/journal.pone.0336046)
Supplement: Attachment — Submitted filename: Questions and Answers 2nd Review.docx [file pone.0336046.s004.docx]

Dear colleagues from PLOSone, Dr. Ody and dear Prof. Kazuyori Yagyu,

Thank you very much for your positive response to my expanded analysis, including text passages. I would also like to thank you for reviewing my text with such care and attention, so that the manuscript can be published as a paper. As in the last review, I have created a Word file called “Questions and Answers 2^nd^ Part” in which I respond to the comments made by the two reviewers.

In addition, I was able to make the data available online in collaboration with my university. The data includes the raw data from the EEG examination, preparatory EEG analysis for N1/P2, as well as readiness potential and clinical data. Further information regarding data processing is stored in a README file. The university has also already created a DOI number (https://doi.org/10.17185/duepublico/84480). It should be noted, however, that the university needs approximately four weeks to make the data available online.

Nevertheless, the university has already generated a link so that the data can be reviewed. The hyperlink is https://nxcl.rds.uni-due.de/s/cprFxrGT7K7Pqms (password: review).

In the following sections, I will provide feedback on each comment made by the two reviewers. If you have any questions regarding the provision of data, please let us know.

Yours sincerely,

Constantin Liermann-Koch

#Reviewer 1:

**Minor issues**

**1# Comment:** Thanks to the authors for addressing my comments. They took them seriously, made appropriate adjustments and the manuscript looks in great shape. I just have a couple of follow up comments.

The statistics for the new analysis that I requested (F(1.92, 105.83) = 2.19, p = 0.0116) does seem to show a significant group difference for Readiness Potential but you wrote in the text that there is no significant difference. Could you please clarify? If there indeed was a group difference for RP and not for LRP, that’s a really interesting result which should be addressed briefly in the discussion.

**1# Answer:** Thank you very much for bringing this to my attention. I have rechecked the statistical analysis and found that I did not enter the results correctly. Primarily, one decimal place too many was added and a 6 was entered instead of a 9. In addition, it was the analysis for interaction between condition and group. The correct data has now been entered. For verification purposes, I have attached the ANOVA with the 3 conditions for RP and LRP as a separated data (“ANOVA with the 3 conditions for RP and LRP”)

.

**2# Comment:** Please replace ‘Condition 1, Condition 2’ etc. in Figure 7 with the actual names of the conditions.

**2# Answer:** Thank you very much for pointing this out. The graphic in Figure 7 has been adjusted accordingly and re-uploaded.

#Reviewer 2:

**Minor revisions:**

**1# Comment:** P3, L77 “Schizophrenia is a neuropsychiatric disorder with an estimated incidence rate of approximately 0.01% with onsets,” Specify the duration of the incidence. I suggest, “Schizophrenia is a neuropsychiatric disorder with an estimated annual incidence rate of approximately 0.01% with onsets,”

**1# Answer:** Thank you very much for suggesting a corrective to this sentence, so that this statement can be properly classified.
